# The Metabolic Role of GRK2 in Insulin Resistance and Associated Conditions

**DOI:** 10.3390/cells10010167

**Published:** 2021-01-15

**Authors:** Daniela Sorriento, Maria Rosaria Rusciano, Valeria Visco, Antonella Fiordelisi, Federica Andrea Cerasuolo, Paolo Poggio, Michele Ciccarelli, Guido Iaccarino

**Affiliations:** 1 Dipartimento di Scienze Biomediche Avanzate, Università Federico II di Napoli, 80131 Napoli, Italy; daniela.sorriento@unina.it (D.S.); antonella.fiordelisi@gmail.com (A.F.); f.andrea_cerasuolo@hotmail.it (F.A.C.); 2 Dipartimento di Medicina, Chirurgia ed Odontoiatria, Università degli Studi di Salerno, 84081 Baronissi, Italy; myra80@gmail.com (M.R.R.); valeriavisco1991@libero.it (V.V.); mciccarelli@unisa.it (M.C.); 3 Unità per lo Studio delle Patologie Aortiche, Valvolari e Coronariche, Centro Cardiologico Monzino IRCCS, 20138 Milan, Italy; paolo.poggio@cardiologicomonzino.it

**Keywords:** GRK2, insulin resistance, diabetes, hypertension, heart failure

## Abstract

Insulin resistance (IRES) is a pathophysiological condition characterized by the reduced response to insulin of several tissues, including myocardial and skeletal muscle. IRES is associated with obesity, glucose intolerance, dyslipidemia, and hypertension, evolves toward type 2 diabetes, and increases the risk of developing cardiovascular diseases. Several studies designed to explore the mechanisms involved in IRES allowed the identification of a multitude of potential molecular targets. Among the most promising, G Protein Coupled Receptor Kinase type 2 (GRK2) appears to be a suitable one given its functional implications in many cellular processes. In this review, we will discuss the metabolic role of GRK2 in those conditions that are characterized by insulin resistance (diabetes, hypertension, heart failure), and the potentiality of its inhibition as a therapeutic strategy to revert both insulin resistance and its associated phenotypes.

## 1. Introduction

**Figure 1:**
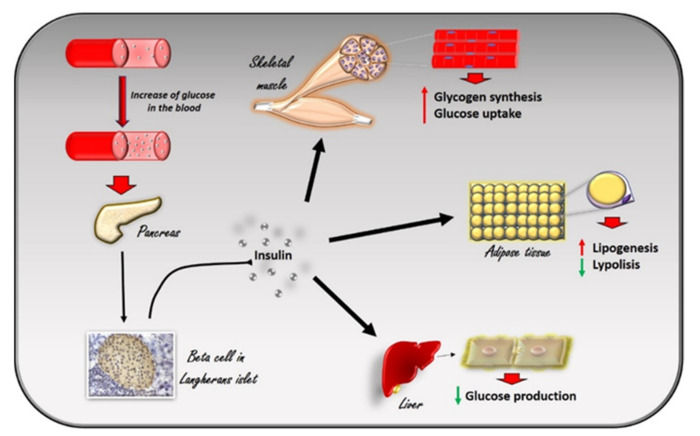
The effects of insulin in response to the increase in glucose levels in the blood. The increase in glucose in the blood stimulates beta cells in the pancreas to produce insulin. Circulating insulin exerts several effects in different tissues. In skeletal muscle, it promotes glucose utilization and storage by increasing glucose transport and glycogen synthesis. In the liver, insulin promotes glycogen synthesis, and inhibits glycogenolysis, gluconeogenesis, and ketogenesis. In white adipocyte tissue, insulin promotes the deposition of triglycerides, inhibits lipolysis, and promotes the absorption of glucose and fatty acids.

Insulin is a peptide hormone produced in the pancreas by the β cells of the Langerhans islets in response to the increase in plasma glucose levels. It induces the rapid absorption of glucose, protein, and fatty acids by tissues for metabolism, storage, and energy production [1] (Figure 1). Alterations in insulin signaling and production, as observed in insulin resistance, significantly impair glucose homeostasis in several pathological conditions, such as diabetes, hypertension, and heart failure. G Protein Coupled Receptor Kinase type 2 (GRK2) has been identified as a mechanism of impaired glucose homeostasis in insulin resistance and its complications. In this review, we will discuss the metabolic role of GRK2 in insulin resistance related conditions.

## 2. Insulin Signaling and the Development of Insulin Resistance

**Figure 2:**
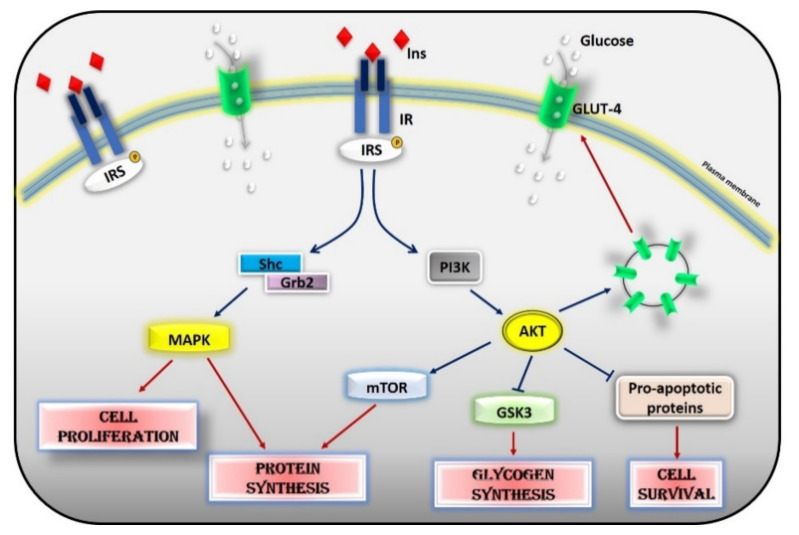
Insulin signaling cascade. Insulin activates the insulin receptor, which phosphorylates IRS in tyrosine residues. IRS, in turn, activates MAPK via SHc/Grb2 and AKT via PI3K. Activated AKT induces glycogen synthesis through inhibition of GSK-3, protein synthesis via mTOR signaling, cell survival through inhibition of several pro-apoptotic agents, and the translocation of GLUT-4 to the cell membrane. Activated MAPK induces cell proliferation and protein synthesis. Abbreviations: INS = Insulin; IR = Insulin Receptor; IRS = Insulin Receptor Substrate; Shc = Src Homology 2 Domain-Containing; Grb2 = Growth factor Receptor-Bound protein 2; MAPK = Mitogen-Activated Protein Kinase; PI3K = Phosphoinositide 3-kinase; AKT = Protein kinase B; mTOR = Mammalian Target of Rapamycin; GSK3 = Glycogen synthase kinase 3; GLUT4 = Glucose Transporter type 4.

Glucose homeostasis is the result of a balance between glucose uptake in organs in the fed state (glycogen synthesis and glucose metabolism) and production of glucose by the liver during fasting (glycogenolysis and gluconeogenesis) and is under the control of a constellation of hormones, from insulin to glucagon, IGF-1, leptin, adiponectin, and adrenergic mechanisms. Insulin is the most potent regulator in the liver, muscle, and adipocytes. Insulin inhibits hepatic glucose output and enhances glucose uptake into skeletal muscle and adipose tissue. The insulin insensitive glucose transporter GLUT-2 in the liver and the insulin-sensitive transporter GLUT-4 in muscle and fat remove glucose from the bloodstream [2]. In muscle and adipose tissue, insulin acts by increasing the number of GLUT 4 at the plasma membrane to induce glucose transport [3,4]. In the liver, the number of GLUT-2 does not change in response to insulin, which in turn regulates the enzymes needed for maintaining glucose cellular disposal [4]. At the molecular level, insulin activates a specific tyrosine kinase receptor, the insulin receptor (IR). Insulin binding to its receptor activates its intrinsic tyrosine kinase activity (Figure 2). IR phosphorylates different substrate adaptors (IRS) in tyrosine residues that form binding sites for intracellular molecules that contain Src-homology 2 (SH2) domains [5,6]. IRS proteins are a family of adaptor proteins whose main role is to convert the tyrosine phosphorylation signal into a lipid kinase signal [5,6]. PI3K is the main active mediator of insulin effects, via the activation of the AKT/PKB cascades. Activated AKT induces glycogen synthesis through inhibition of GSK-3, protein synthesis via mTOR signaling, cell survival through inhibition of several pro-apoptotic agents, and autophagy by inhibiting FoxO transcription factors. AKT also regulates the translocation of the insulin-sensitive glucose transporter GLUT-4 to the cell membrane within muscle and fat cells for the extraction of glucose [5]. Insulin also acts through the mitogen-activated protein kinase (MAPK) pathway, by phosphorylating the IRS proteins, Gab1, and Shc [7]. The activation of the MAPK cascade is associated with the proliferative effects of insulin [7,8].

Phosphorylation of serine residues in IRS1 could exert both positive and negative effects on insulin signaling [9]. Data from previous studies show a time-dependent regulation of phosphorylative events leading to early activation and inhibition of the insulin pathway through IRS-1. Positive regulatory sites are early phosphorylated to activate the insulin signaling pathway. On the contrary, the inhibitory sites are phosphorylated later to turn off the insulin signaling and disrupt the interaction between IRS1 and IR, and to promote IRS1 degradation [10]. Insulin signaling is therefore strictly dependent on a correct balance between the phosphorylation of positive and negative sites, which is altered in pathophysiological conditions. Insulin resistance (IRES) occurs when the excess of glucose in the blood induces an increase in insulin release that makes the target organs resistant to its action. Insulin resistance is a hallmark of two pathological conditions: metabolic syndrome and type 2 diabetes. Indeed, it is associated with obesity, glucose intolerance, dyslipidemia, and hypertension, which can successively progress to type 2 diabetes and cardiovascular diseases. It also increases the risk of cancer [11]. The precise pathogenetic mechanism of IRES is not yet clear but several molecular mechanisms (oxidative stress, inflammation, insulin receptor mutation, ER stress, mitochondrial dysfunction) have been demonstrated to be involved [10,12,13]. Chronic inflammation in targeted tissues was shown to contribute to altering the metabolic state, both locally via autocrine/paracrine cytokine signaling and systemically via endocrine cytokine signaling [14]. Reactive oxygen species (ROS) cause insulin resistance in the peripheral tissues by decreasing the expression of GLUT4 in the plasma membrane [15]. Then, mitochondrial alteration and obesity contribute to propagating oxidative stress [15]. In the skeletal muscle, mitochondrial dysfunction causes the development of insulin resistance in different ways: (1) by activating several protein kinases, such as Protein kinase C (PKC) or c-Jun N-terminal kinase (JNK), which inhibit IRS-1 signaling by phosphorylation; (2) by reducing the expression levels of IRS-1 [16]; and (3) by inducing ER stress through excessive cytosolic calcium levels [17]. Several factors can influence insulin sensitivity such as obesity and fat distribution [18,19], age and sex [19], genetics, exercise [20], dietary nutrients [21], and hormones (glucagon, epinephrine, cortisol, leptin, growth hormones, sex hormones, amylin). Obesity is associated with an increased risk of developing insulin resistance. Indeed, the production of many mediators of glucose metabolism by the adipose tissue (Non Esterified Fatty Acids, glycerol, leptin, and adiponectin; proinflammatory cytokines) are increased in obesity [22]. In addition, mutations in genes involved in the insulin signaling cascade (insulin receptor, PI3-kinase and AKT) cause severe insulin resistance [2,23] as well as mutations and polimorphisms in genes that predispose to obesity (melanocortin-4 receptor, leptin receptor, peroxisome proliferator-activated receptor PPARg).

Insulin signaling is very complex and involves a large number of molecules that are all crucial to activate insulin-dependent biological processes. Therefore, several events could regulate insulin effects and sensitivity, such as protein degradation and synthesis, phosphorylative events, post-translational modifications, as well as the inhibition of single components at different steps of the long insulin-dependent cascade. In this context, the G Protein-Coupled Receptor Kinase 2 (GRK2) is considered a key regulator of metabolic processes and, in particular, of glucose metabolism.

## 3. GRK2 Affects Insulin Signaling

G Protein-Coupled Receptor Kinases (GRK) are known to phosphorylate G Protein Coupled Receptors GPCRs on the plasma membrane to turn off their intracellular signaling [24,25]. Therefore, GRKs exert a critical role in both physiological states and pathological conditions that are mainly associated with alterations of the adrenergic signaling. Besides this classical effect, GRKs are also known to inactivate non-GPCR receptors, including tyrosine kinase receptors, by phosphorylative events [24]. Moreover, they can interact with and regulate the activity of several cytosolic substrates [26,27,28]. Therefore, GRKs are involved in the regulation of key processes within the cell and are, consequently, potential therapeutic targets [26,29]. Among GRKs, GRK2 is known to regulate cell metabolism [29,30,31]. GRK2 shuttles among the different subcellular compartments following the energetic requests of the cell [32,33]. In particular, we showed that GRK2 localizes in mitochondria and regulates critical mitochondrial processes, such as ATP production and mitochondrial biogenesis [32]. The overexpression of the kinase favors such processes preventing ATP loss after hypoxia/reperfusion [32] and mitochondrial damage in response to acute exposure to ionizing radiation [34]. Despite energy metabolism, the suggestion of GRK2 involvement in cell metabolism comes from the demonstration that insulin induces an up-regulation of the kinase expression levels and the association between GRK2 and IRS1 [35]. GRK2 phosphorylates IRS1 in serine/threonine and inhibits the activation of the downstream signaling. In particular, GRK2 inhibits the phosphorylation of IRS1 in Tyr612, blocking the insulin signaling, and induces its phosphorylation in Ser307, promoting IRS degradation [35,36]. These results are in line with the reciprocal relationship between these two sites of IRS phosphorylation that is described in previous reports [37,38]. Chronic stimulation of beta-adrenergic receptors (**β**AR) is involved in the pathogenesis of insulin resistance. In cells overexpressing the **β**AR, GRK2 accumulates in the plasma membrane and inhibits IRS1 activation and glucose uptake in response to insulin. The selective inhibition of the kinase potentiates insulin signaling both in vitro and in vivo suggesting that GRK2 mediates adrenergic insulin resistance while its inhibition increases insulin sensitivity [35]. These observations point to GRK2 as a potential target, also considering that conditions associated with insulin resistance (diabetes, hypertension, or chronic activation of β adrenergic receptor), are all characterized by elevated kinase levels. Indeed, GRK2 expression is increased in key tissues in different experimental models of insulin resistance, and its downregulation ameliorates the alterations of glucose homeostasis and insulin signaling in response to different insults [39]. Recently, the role of GRK2 in myeloid cells has also been investigated. Vila-Bedmar and colleagues suggest that the downregulation of GRK2 in these cells reduces the pro-inflammatory features of macrophages and prevents high fat diet-induced metabolic alterations [40]. GRK2 expression levels, as well as its subcellular localization and activity, are finely regulated. Several molecules are involved in regulating different steps of kinase transcription, mRNA regulation and translation, protein maturation, localization, activation, and degradation, as extensively described in the review from Penela and colleagues [41]. Here, we will discuss the recent findings on the metabolic role of GRK2 in insulin resistance-related conditions, such as diabetes, hypertension, and heart failure.

## 4. Type 2 Diabetes

Diabetes is the result of a cluster of metabolic alterations including insulin resistance, lower insulin secretion, or increased glucagon secretion leading to hyperglycemia. Based on insulin availability, diabetes can be classified into Type I insulin-dependent, Type II insulin-independent, Idiopathic diabetes and Gestational diabetes, and Type I and Type II diabetes are the most common ones. Type 1 diabetes (T1D) is an autoimmune disorder characterized by the rapid destruction of pancreatic beta cells. For T1D, therapy with insulin replacement is needed since the autoimmune attack leads to an absolute lack of insulin production. Type 1 diabetes occurs mostly in young people except for LADA (Latent Autoimmune Diabetes of the Adult) in which the autoimmune attack is slow and less severe with clinical signs appearing at an older age. Type 2 diabetes (T2D) is the most common form of diabetes. It is characterized by insulin resistance of the peripheral tissues and inadequate insulin production. The disorder of carbohydrate metabolism damages various target organs and tissues, mainly the eye (retinopathy), kidney (nephropathy), nerves (neuropathy), arteries (vasculopathy), and heart (heart disease) [42]. These organ damages are often worsened by the concurrent presence of dyslipidemia and high blood pressure, which are, together with thrombophilia, chronic inflammation, oxidative stress and endothelial dysfunction, mechanisms of insulin resistance [43]. T2D is, therefore, a multifactorial disorder affecting several organs and tissues with a complex mechanism [44,45]. The anatomic trilogy (pancreas, muscle, and liver) is the active player in the development of glucose intolerance in Type 2 diabetic patients [46] together with the fat cell, gastrointestinal tract, α-cell, kidney, and brain [46]. Both genetic and environmental factors (obesity, overeating, sedentary lifestyle, stress, aging) predispose to T2D [47]. Genetic factors, including IR and IRS1 gene polymorphisms [48], directly affect insulin signaling. In addition, polymorphisms of other genes, such as the beta 3 adrenergic receptor gene [49] and the uncoupling protein gene [50], lead to insulin resistance by favoring abdominal obesity. Several molecular mechanisms, including apoptosis, oxidative stress, mitochondrial dysfunction, and inflammation, are all involved in insulin resistance [44,51]. Pre-clinical and clinical studies show the role of oxidative stress in the development of diabetes. Free radicals derive from glucose oxidation, nonenzymatic glycation of proteins, and increased lipid peroxidation. The increase in free radicals together with reduced scavenging leads to the development of insulin resistance and diabetes [52]. Mitochondria play a role as the main source of oxidative stress. Alterations of the mitochondrial number, morphology, and function are described in insulin-resistant muscles [53]. Inflammation has been associated with type 2 diabetes and its complications, as both a cause and a consequence. Indeed, low-grade chronic inflammation is the trigger of insulin resistance in type 2 diabetes and the continuous release of pro-inflammatory mediators favors the development of the associated conditions [54]. Several pharmacologic approaches are currently in use for the management of diabetes, including insulin injections, metformin, Sulfonylureas, peroxisome proliferator-activated receptor γ activators, Dipeptidyl peptidase-4 (DPP4) inhibitors, Glucagon-like peptide 1 (GLP-1) analogues, and Sodium–glucose co-transporter-2 (SGLT2) inhibitors [55]. Given the complexity of the pathogenic mechanism, multiple therapeutic approaches are required for diabetes management and combined therapy is preferred. In this context, the discovery of a common molecular target for the treatment of all clinical signs is the current goal of research in the field.

**Figure 3:**
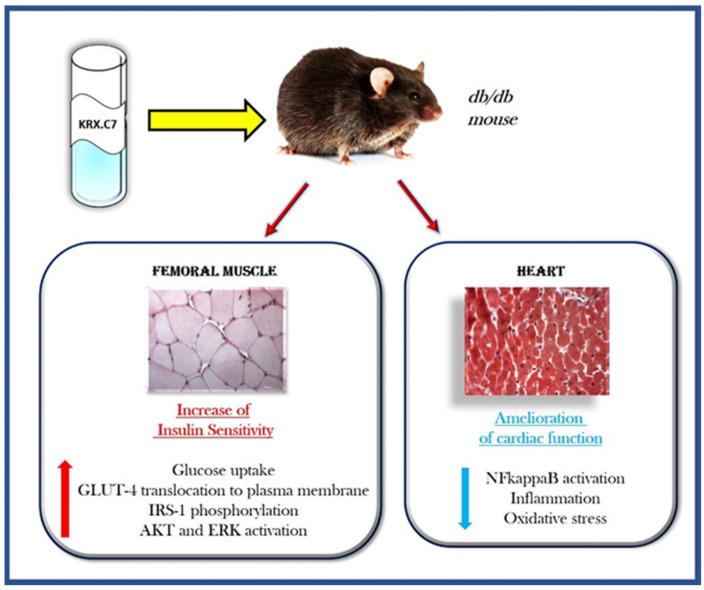
The double effect of GRK2 inhibition in diabetic mice. In db/db mice, the treatment with KRX-C7, a selective inhibitor of GRK2, increases insulin sensitivity by affecting the insulin signaling cascade. Indeed, it induces the phosphorylation of IRS-1 and the activation of AKT and MAPK signaling. This induces the translocation of GLUT-4 to the plasma membrane and increases glucose uptake. Abbreviations: KRX-C7 = cyclic peptide inhibitor of GRK2; GLUT4 = Glucose Transporter type 4; IRS = Insulin Receptor Substrate; AKT = Protein kinase B; ERK = Extracellular signal-Regulated Kinase; NFkappaB = Nuclear Factor kappa-light-chain-enhancer of activated B cells.

Several studies support the role of GRK2 in the development and progression of diabetes. Insulin induces an up-regulation of the kinase, mainly favoring its translocation on the plasma membrane to regulate IR signaling [35]. However, the first evidence comes from the demonstration that GRK2 inhibits the insulin signaling cascade, leading to glucose transport in 3T3-L1 adipocytes [56]. Indeed, the kinase overexpression inhibits insulin-stimulated GLUT-4 translocation and glucose uptake while its deletion exerts the opposite effect. GRK2 directly binds Gαq/11 through the RGS domain and inhibits insulin-induced activation of the Gαq/11 pathway. Moreover, in a mouse model of obesity and insulin resistance, the inducible GRK2 ablation reverts the insulin-resistant and obese phenotype [57]. Besides its ability to regulate glucose homeostasis, GRK2 also regulates other molecular mechanisms that contribute to the development of diabetes and its complications, such as endothelial and cardiac dysfunction [58,59]. Indeed, GRK2 is an ubiquitary protein and can be found in all insulin-sensitive cell types (hepatocytes, endothelial cells, cardiomyocytes). GRK2 is a key contributor to vascular endothelial dysfunction in diabetes by inhibiting the AKT/eNOS pathway in endothelial cells [60]. Findings from animal models of diabetes show that GRK2 silencing in the liver significantly improves glucose homeostasis, but also ameliorates diabetes-dependent endothelial dysfunction [58]. GRK2 deletion in the liver improves the impaired endothelial AKT/eNOS-dependent signal activation in type 2 diabetic aortas [58]. This double effect hypothesizes that the downregulation of GRK2 levels in the liver ameliorates the impaired endothelial signaling and vascular responses in type 2 diabetic aortas by improving glucose homeostasis and insulin sensitivity in the liver. The ability of GRK2 to ameliorate diabetic endothelial function is also demonstrated in other studies. In ob/ob mice, GRK2 inhibition, or silencing, prevented the reduction in insulin-induced relaxation by increasing the activation of insulin signaling [61]. Functional crosstalks between different cell types which are orchestrated by GRK2 also involve the immune cells. Indeed, the inhibition of GRK2, by the HJ-loop custom peptide KRX-C7, counteracts the inflammatory phenotype of the diabetic heart by blocking the NFkappaB dependent pathway in db/db mice [59]. These observations suggest that GRK2 is a potential therapeutic target for the treatment of diabetes and its associated phenotypes due to its pleiotropy in different cells and tissues. To date, most treatments for diabetes are not effective on its associated phenotypes, such as diabetic cardiomyopathy, even favoring the progression of the disease in some cases. The inhibition of GRK2 fits well in this context, being able to both reduce insulin resistance and diabetes-dependent phenotypes [59] (Figure 3).

Indeed, GRK2 expression in peripheral lymphocytes of patients with heart failure correlates with kinase levels in the heart and is associated with myocardial failure [62,63]. These data support the concept that GRK2 not only regulates intracellular signaling in pathological conditions but can also be used as a biomarker of disease progression. Such a correlation also exists in diabetes. Indeed, elevated GRK2 levels in lymphocytes is associated with diabetes [64], and kinase levels increase in the myocardial tissue and the PBMCs at the early stage of diabetes in db/db mice [65]. These findings support the idea that GRK2 could also be a potential biomarker for diabetes.

## 5. Hypertension

Hypertension is a complex and multifactorial disease and represents one of the major causes of mortality and morbidity in industrialized countries [66]. It also represents a common risk factor for many cardiovascular diseases, including stroke, coronary artery disease, atrial fibrillation, and peripheral vascular disease, and also significantly contributes to the incidence of heart failure [67].

**Figure 4:**
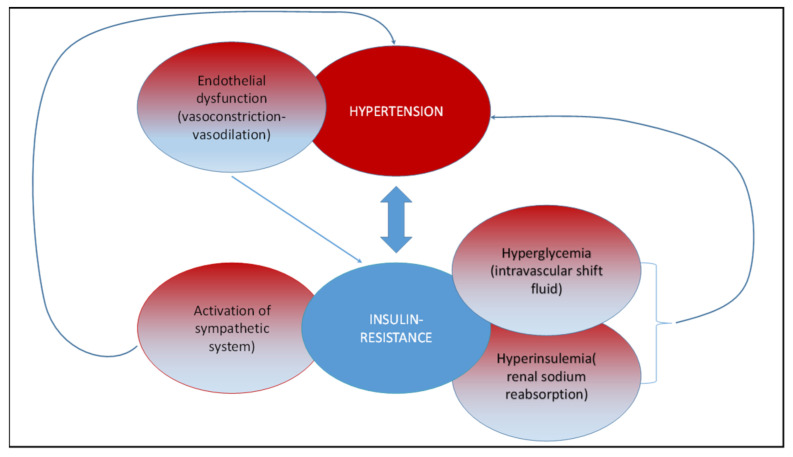
Network linking hypertension and insulin resistance. Hypertension and insulin resistance are strictly connected. Insulin resistance induces hyperglycemia, compensative hyperinsulinemia and activation of the sympathetic system, which, in turn, favors the development of a hypertensive state. On the other hand, hypertension is characterized by endothelial dysfunction, which induces insulin resistance.

Several studies reported that hypertension and insulin resistance are strictly connected [68,69,70,71,72]. Insulin resistance is a pathological condition in which target tissues fail to respond to insulin stimulation, leading to hyperglycemia and compensative hyperinsulinemia [73]. Hyperglycemia acts on electrolytic balance, driving a fluid shift from the intracellular to the extracellular compartment. The body water and solutes redistribution lead to plasma volume expansion and blood pressure (BP) elevation [74]. Hyperinsulinemia directly elevates BP via several mechanisms: increased renal sodium reabsorption, activation of the sympathetic nervous system, alteration of transmembrane ion transport, and hypertrophy of resistance vessels. Hypertension and insulin resistance represent the main actors of metabolic syndrome, a complex and multifactorial disease, also including obesity, glucose intolerance, and dyslipidemia, which lead to a dramatic increase in cardiovascular morbidity and mortality [75,76,77]. A complex network of different factors has been proposed to explain the underlying mechanisms for the development of hypertension in the metabolic syndrome, including: visceral/central obesity, insulin resistance, sympathetic overactivity [78], oxidative stress, endothelial dysfunction, and activated renin-angiotensin system [79,80] (Figure 4).

Currently, the pharmacological therapy used to treat essential hypertension is focused on the regulation of vascular resistance through the receptor antagonism of hormones such as catecholamines and angiotensin II. GRK2 is involved in the regulation of several processes such as mitochondrial homeostasis, aldosterone secretion, renal regulation of sodium excretion, vasoconstriction in response to VSM α1AR stimulation, and endothelial dysfunction, which can all contribute to the development of the hypertensive phenotype. The presence of an altered expression in GRK2 is involved in both human and animal models of hypertension [81,82,83,84]. In particular, it has been shown that in lymphocytes of hypertensive patients, the activity of GRK2 is significantly increased and this leads to beta-adrenergic desensitization typical of the hypertensive state [82]. These findings were confirmed in another study where the authors demonstrated that the increased activity of GRK2 is causative for the impaired beta adrenergic receptor (βAR) vasodilation in hypertensive patients [84]. Moreover, in spontaneously hypertensive rats (SHRs) aged 10 and 15 weeks, GRK2 overexpression in both lymphocytes and aortic vascular smooth muscle cells is associated with the impairment of beta-adrenergic-mediated stimulation of adenylyl cyclase activity and beta AR-mediated vasodilation [85]. The metabolic role of GRK2 is indeed suggested by the animal model of hypertension, such as SHR, where the increased level of the kinase fosters IRES [35]. Furthermore, modulation of the GRK2 level represents a potential target for the treatment of metabolic disorders [86]. Increased mitochondrial production of reactive oxygen species (ROS) has been also associated with hypertension [34,87,88]. GRK2 participates in mitochondrial dynamics and, consequently, is involved in ROS production. In HEK-293 cells, the GRK2 removal by siRNA impinges on mitochondrial mass, morphology, and function after exposure to an acute stimulus such as ionizing radiation (IRa). siRNA-GRK2 treated cells showed alteration in their mitochondrial ultrastructure at the basal level, before IRa exposure; moreover, a progressive ROS accumulation has been observed. Otherwise, the overexpression of GRK2 preserves mitochondrial morphology and structure, showing ROS accumulation in basal condition but with no modification at 3 h and 8 h post-IRa [34]. Accordingly, selective removal of GRK2 from endothelium also enhanced mitochondrial ROS, leading to expression of cytokines associated with an inflammatory state of the vessel wall, and the treatment with ROS scavengers reduced aorta and lung inflammation and restored vasomotor responses [88]. Overall, while excessive GRK2 signaling appears to both contribute to the establishment of IRES, HTN and metabolic syndrome, the absence of GRK2 also affects endothelial homeostasis, demonstrating the physiological role of the kinase.

## 6. Heart Failure

Heart failure (HF) is a complex syndrome, where structural and/or functional cardiac alterations, reduced cardiac output and/or elevated intracardiac pressures both at rest as well as during stress variably coexist [89]. While the management of HF has made significant progress [90,91], this condition still represents the leading cause of disability and mortality in the world [92]. Heart failure could be considered as a global pandemic of modern society with increasing rates in prevalence, incidence, mortality, and morbidity, thus requiring many efforts to understand its pathophysiology in order to develop new and effective treatments. Consequently, the discovery and implementation of specific targets that might aid in the diagnosis, prognosis, risk stratification, and treatment of HF patients could be a critical point in contemporary clinical practice [93]. The primary step of HF is the acute and chronic myocyte injury caused by several factors such as coronary artery disease, uncontrolled arterial hypertension, and diabetes mellitus; collectively, these foster an adverse and progressive myocardial remodeling [94,95] through upregulated neurohumoral activation, impaired intracellular calcium cycling, accelerated apoptosis of cardiac myocytes, imbalance in the formation and the breakdown of the extracellular matrix [96]. Indeed, patients remain asymptomatic for a long time. Nevertheless, the chronic activation of compensatory neurohormonal mechanisms affects the cardiac structure and function, leading to a progressive clinical worsening. They encompass the activation of the renin-angiotensin-aldosterone system (RAAS), arginine-vasopressin system, and kallikrein-kininogen-kinin system, as well as the activation of the natriuretic peptides system, neprilysin signaling pathway, endothelin pathway, and cytokine systems, and the upregulation of adrenergic/SNS pathways [97,98,99,100]. GRK2 has been demonstrated to be pivotal in the regulation of cardiac functions, including beta adrenergic receptor signaling, contractility, and myocardial energy production and expenditure.

**Figure 5:**
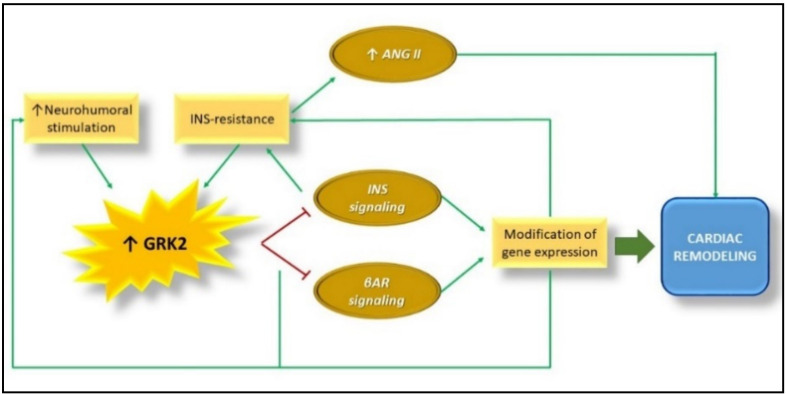
GRK2 up-regulation, Insulin resistance, and maladaptive cardiac remodeling in heart failure. Several studies showed that myocardial GRK2 activity and expression is increased in the failing heart. This promotes desensitization and downregulation of βAR and inhibits INS signaling, causing the modification of hypertrophic gene expression and, consequently, cardiac remodeling. The alterations of INS signaling cause Insulin resistance that in turn induces Ang II-dependent cardiac remodeling. Abbreviations: GRK2 = G Protein coupled Receptor Kinase type 2; βAR = beta adrenergic receptor; INS = Insulin; ANG II = Angiotensin II.

In HF, clinical variability reflects biochemical complexity and alterations in various signals: the failing cardiomyocyte has adrenergic incompetence and loses metabolic plasticity [101], with increased ROS production and impaired calcium handling [102]. GRKs has multiple roles in cardiovascular physiopathology and works in concert with beta-arrestins to desensitize, internalize, and ultimately down-regulate GPCRs [103]; additionally, beta-arrestins can independently signal from G protein activation [104]. The correct balance of these effects is, therefore, critical for the right geometry and remodeling of the injured heart. GRK2 regulates insulin signaling [35] through serine phosphorylative events [30]. Excessive GRK2 up-regulation inhibits insulin signaling and glucose extraction due to a time-dependent insulin-stimulated association of GRK2 with IRS1, leading to IRS1 serine phosphorylation and inactivation [35] and, consequently, to IRES in HF. In this sense, GRK2 inhibition improves insulin sensitivity, suggesting a new therapeutic strategy for the treatment of IRES and T2DM [30]. Chronic hyperinsulinemia associated with whole-body IRES can stimulate angiotensin II-induced pathological hypertrophy [105]. In addition, it inhibits beta adrenergic receptor-dependent cardiac contractility through the downregulation of beta 2 adrenergic receptors due to GRK2 kinase activity [106,107] (Figure 5).

Inversely, the total deletion of the kinase interferes with the correct development of the CV system [108]. The GRK2 knockdown in myocytes predisposes to a prevalent eccentric remodeling upon a chronic beta adrenergic stimulation [109]. These data argue strongly that the role of GRK2 in the pathogenesis of HF is due, at least in part, to negative alterations in cardiac metabolism. These defects in cardiac metabolism are associated with GRK2 kinase activity. Indeed, mice with cardiac-specific overexpression of GRK2 (TgGRK2), as it occurs in HF, show an impaired fatty acid uptake rate and energetics, due to the ability of GRK2 to phosphorylate CD36 [110,111]. Moreover, the phosphorylation of GRK2 itself in Serine 670 is associated with cardiomyocyte death post-ischemia-reperfusion injury and this phenotype is rescued in mice with a GRK2 S670A knock-in mutation [112]. Recently, the effects of GRK2 inhibition on cardiac metabolism and mitochondrial function have been evaluated in a mice model of HF by the administration of a cyclic peptide inhibitor, C7, already tested for its effectiveness and selectivity in several experimental models [59,108,113]. The inhibition of GRK2 ameliorates alterations in cardiac metabolism and mitochondrial function. Indeed, C7 significantly improves cardiac lipid metabolism, and recovers mitochondrial morphology, mitochondrial biogenesis, mitochondrial respiration, and ATP production [114]. Accordingly, previous works, employing different strategies of GRK2 inhibition, demonstrated that reducing the activity of this kinase restores myocardial function at the biochemical and contractile levels [100]. These data support the idea that the inhibition of GRK2 could be a useful strategy to correct alterations of cardiac metabolic state in both physiological and pathological conditions.

## 7. Conclusions

A growing number of molecular mechanisms could hypothetically be targeted through pharmacological approaches for the treatments of insulin resistance and associated pathologies. Among the different molecules, GRK2 certainly stands out, because molecular complexity and mechanistic implications in different contests are well demonstrated and documented (Table 1). Interestingly, several drugs currently in use to control glycemia, such as SGLT-2 inhibitors and Glucagon-like peptide-1 (GLP-1), can also improve cardiac health due to their ability to act on different processes within the cell (endothelial dysfunction, inflammation, Ca^2+^ overload, mitochondrial dysfunction) [115,116,117]. In this context, the inhibition of GRK2 could be a potential therapeutic strategy for those conditions correlated with insulin resistance due to the pleiotropic effects of the kinase. To date, the impact of a GRK2 inhibitor on human therapy cannot be foreseen despite the evidence that short peptides derived from the HJ loop of GRK2 [118] possess promising metabolic effects in animal models of Type 2 diabetes and IRES, as well as in endothelial function and heart failure.

## Figures and Tables

**Table 1:** Summary of GRK2 effects in insulin resistance and its associated phenotypes.

Obesity/Insulin Resistance Diabetes	Hypertension	Heart Failure
GRK2 phosphorylates IRS1 and inhibits the downstream signaling [35,36] GRK2 inhibits insulin signaling in 3T3-L1 adipocytes [56] GRK2 inhibits insulin-induced GLUT-4 translocation and glucose uptake [56] GRK2 inhibits the AKT-eNOS pathway in endothelial cells, contributing to endothelial dysfunction diabetes [60] In ob/ob mice GRK2 inhibition activates insulin signaling [57,61] GRK2 silencing in the liver improves glucose homeostasis, ameliorating endothelial function [58] GRK2 inhibits the NFkappaB pathway, counteracting inflammation in db/db mice [59]	GRK2 upregulation in lymphocytes of hypertensive patients induces beta adrenergic desensitization [81,82] GRK2 impairs betaAR vasodilation in hypertensive patients [84] Increased GRK2 levels in lymphocytes and aortic SMC impairs adenylyl cyclase activity and betaAR vasodilation [85] GRK2 regulates blood pressure, promoting epithelial Na^+^ channel activity [85] GRK2 promotes ROS production [87,88]	GRK2 promotes IRES in HF by inactivating IRS1 [35] GRK2 downregulation promotes eccentric cardiac remodeling [109] Overexpression of GRK2 impairs Fatty Acid uptake [110,111] Inhibition of GRK2 improves cardiac metabolism [1,14]

## Data Availability

Not Applicable.

## References

[1] Petersen M.C., Shulman G.I. (2018). Mechanisms of Insulin Action and Insulin Resistance. Physiol. Rev..

[2] Czech M.P. (2017). Insulin action and resistance in obesity and type 2 diabetes. Nat. Med..

[3] Dugani C.B., Klip A. (2005). Glucose transporter 4: Cycling, compartments and controversies. EMBO. Rep..

[4] Chadt A., Al-Hasani H. (2020). Glucose transporters in adipose tissue, liver, and skeletal muscle in metabolic health and disease. Pflug. Arch..

[5] Haeusler R.A., McGraw T.E., Accili D. (2018). Biochemical and cellular properties of insulin receptor signalling. Nat. Rev. Mol. Cell Biol..

[6] Boucher J., Kleinridders A., Kahn C.R. (2014). Insulin Receptor Signaling in Normal and Insulin-Resistant States. Cold Spring Harb. Perspect. Biol..

[7] Kong L., Wang Q., Jin J., Xiang Z., Chen T., Shen S., Wang H., Gao Q., Wang Y. (2017). Insulin resistance enhances the mitogen-activated protein kinase signaling pathway in ovarian granulosa cells. PLoS ONE.

[8] Biddinger S.B., Kahn C.R. (2006). From mice to men: Insights into the insulin resistance syndromes. Annu. Rev. Physiol..

[9] Weigert C., Kron M., Kalbacher H., Pohl A.K., Runge H., Haring H.U., Schleicher E., Lehmann R. (2008). Interplay and effects of temporal changes in the phosphorylation state of serine-302, -307, and -318 of insulin receptor substrate-1 on insulin action in skeletal muscle cells. Mol. Endocrinol..

[10] Tanti J.F., Jager J. (2009). Cellular mechanisms of insulin resistance: Role of stress-regulated serine kinases and insulin receptor substrates (IRS) serine phosphorylation. Curr. Opin. Pharm..

[11] Vigneri R., Goldfine I.D., Frittitta L. (2016). Insulin, insulin receptors, and cancer. J. Endocrinol. Investig..

[12] Sesti G. (2006). Pathophysiology of insulin resistance. Best Pract. Res. Clin. Endocrinol. Metab..

[13] Yaribeygi H., Farrokhi F.R., Butler A.E., Sahebkar A. (2019). Insulin resistance: Review of the underlying molecular mechanisms. J. Cell Physiol..

[14] De Luca C., Olefsky J.M. (2008). Inflammation and insulin resistance. FEBS Lett..

[15] Hurrle S., Hsu W.H. (2017). The etiology of oxidative stress in insulin resistance. Biomed. J..

[16] Ryu H.S., Park S.Y., Ma D., Zhang J., Lee W. (2011). The induction of microRNA targeting IRS-1 is involved in the development of insulin resistance under conditions of mitochondrial dysfunction in hepatocytes. PLoS ONE.

[17] Lim J.H., Lee H.J., Ho Jung M., Song J. (2009). Coupling mitochondrial dysfunction to endoplasmic reticulum stress response: A molecular mechanism leading to hepatic insulin resistance. Cell Signal..

[18] Kahn S.E., Prigeon R.L., Schwartz R.S., Fujimoto W.Y., Knopp R.H., Brunzell J.D., Porte D. (2001). Obesity, body fat distribution, insulin sensitivity and Islet beta-cell function as explanations for metabolic diversity. J. Nutr..

[19] Karakelides H., Irving B.A., Short K.R., O’Brien P., Nair K.S. (2010). Age, obesity, and sex effects on insulin sensitivity and skeletal muscle mitochondrial function. Diabetes.

[20] Borghouts L.B., Keizer H.A. (2000). Exercise and insulin sensitivity: A review. Int. J. Sports Med..

[21] Galgani J.E., Uauy R.D., Aguirre C.A., Diaz E.O. (2008). Effect of the dietary fat quality on insulin sensitivity. Br. J. Nutr..

[22] Kahn S.E., Hull R.L., Utzschneider K.M. (2006). Mechanisms linking obesity to insulin resistance and type 2 diabetes. Nature.

[23] Parker V.E., Savage D.B., O’Rahilly S., Semple R.K. (2011). Mechanistic insights into insulin resistance in the genetic era. Diabet. Med..

[24] Ribas C., Penela P., Murga C., Salcedo A., Garcia-Hoz C., Jurado-Pueyo M., Aymerich I., Mayor F. (2007). The G protein-coupled receptor kinase (GRK) interactome: Role of GRKs in GPCR regulation and signaling. Biochim. Biophys. Acta.

[25] Gambardella J., Sorriento D., Bova M., Rusciano M., Loffredo S., Wang X., Petraroli A., Carucci L., Mormile I., Oliveti M. (2020). Role of Endothelial G Protein-Coupled Receptor Kinase 2 in Angioedema. Hypertension.

[26] Penela P., Murga C., Ribas C., Lafarga V., Mayor F. (2010). The complex G protein-coupled receptor kinase 2 (GRK2) interactome unveils new physiopathological targets. Br. J. Pharm..

[27] Sorriento D., Ciccarelli M., Santulli G., Campanile A., Altobelli G.G., Cimini V., Galasso G., Astone D., Piscione F., Pastore L. (2008). The G-protein-coupled receptor kinase 5 inhibits NFkappaB transcriptional activity by inducing nuclear accumulation of IkappaB alpha. Proc. Natl. Acad. Sci. USA.

[28] Sorriento D., Santulli G., Ciccarelli M., Maione A.S., Illario M., Trimarco B., Iaccarino G. (2018). The Amino-Terminal Domain of GRK5 Inhibits Cardiac Hypertrophy through the Regulation of Calcium-Calmodulin Dependent Transcription Factors. Int. J. Mol. Sci..

[29] Sorriento D., Gambardella J., Fiordelisi A., Iaccarino G., Illario M. (2019). GRKs and beta-Arrestins: “Gatekeepers” of Mitochondrial Function in the Failing Heart. Front. Pharm..

[30] Ciccarelli M., Cipolletta E., Iaccarino G. (2012). GRK2 at the control shaft of cellular metabolism. Curr. Pharm. Des..

[31] Sorriento D., Ciccarelli M., Santulli G., Illario M., Trimarco B., Iaccarino G. (2014). Trafficking GRK2: Cellular and Metabolic consequences of GRK2 subcellular localization. Transl. Med. UniSa.

[32] Fusco A., Santulli G., Sorriento D., Cipolletta E., Garbi C., Dorn G.W., Trimarco B., Feliciello A., Iaccarino G. (2012). Mitochondrial localization unveils a novel role for GRK2 in organelle biogenesis. Cell Signal..

[33] Sorriento D., Fusco A., Ciccarelli M., Rungi A., Anastasio A., Carillo A., Dorn G.W., Trimarco B., Iaccarino G. (2013). Mitochondrial G protein coupled receptor kinase 2 regulates proinflammatory responses in macrophages. FEBS Lett..

[34] Franco A., Sorriento D., Gambardella J., Pacelli R., Prevete N., Procaccini C., Matarese G., Trimarco B., Iaccarino G., Ciccarelli M. (2018). GRK2 moderates the acute mitochondrial damage to ionizing radiation exposure by promoting mitochondrial fission/fusion. Cell Death Discov..

[35] Cipolletta E., Campanile A., Santulli G., Sanzari E., Leosco D., Campiglia P., Trimarco B., Iaccarino G. (2009). The G protein coupled receptor kinase 2 plays an essential role in beta-adrenergic receptor-induced insulin resistance. Cardiovasc. Res..

[36] Ciccarelli M., Chuprun J.K., Rengo G., Gao E., Wei Z., Peroutka R.J., Gold J.I., Gumpert A., Chen M., Otis N.J. (2011). G protein-coupled receptor kinase 2 activity impairs cardiac glucose uptake and promotes insulin resistance after myocardial ischemia. Circulation.

[37] Shahid G., Hussain T. (2007). GRK2 negatively regulates glycogen synthesis in mouse liver FL83B cells. J. Biol. Chem..

[38] Aguirre V., Werner E.D., Giraud J., Lee Y.H., Shoelson S.E., White M.F. (2002). Phosphorylation of Ser307 in insulin receptor substrate-1 blocks interactions with the insulin receptor and inhibits insulin action. J. Biol. Chem..

[39] Garcia-Guerra L., Nieto-Vazquez I., Vila-Bedmar R., Jurado-Pueyo M., Zalba G., Diez J., Murga C., Fernandez-Veledo S., Mayor F., Lorenzo M. (2010). G protein-coupled receptor kinase 2 plays a relevant role in insulin resistance and obesity. Diabetes.

[40] Vila-Bedmar R., Cruces-Sande M., Arcones A.C., Willemen H., Prieto P., Moreno-Indias I., Diaz-Rodriguez D., Francisco S., Jaen R.I., Gutierrez-Repiso C. (2020). GRK2 levels in myeloid cells modulate adipose-liver crosstalk in high fat diet-induced obesity. Cell Mol. Life Sci..

[41] Penela P., Ribas C., Sanchez-Madrid F., Mayor F. (2019). G protein-coupled receptor kinase 2 (GRK2) as a multifunctional signaling hub. Cell Mol. Life Sci..

[42] Nathan D.M., Group D.E.R. (2014). The diabetes control and complications trial/epidemiology of diabetes interventions and complications study at 30 years: Overview. Diabetes Care.

[43] Oguntibeju O.O. (2019). Type 2 diabetes mellitus, oxidative stress and inflammation: Examining the links. Int. J. Physiol. Pathophysiol. Pharm..

[44] Kahn S.E., Cooper M.E., Del Prato S. (2014). Pathophysiology and treatment of type 2 diabetes: Perspectives on the past, present, and future. Lancet.

[45] Scheen A.J. (2003). Pathophysiology of type 2 diabetes. Acta Clin. Belg..

[46] Defronzo R.A. (2009). From the triumvirate to the ominous octet: A new paradigm for the treatment of type 2 diabetes mellitus. Diabetes.

[47] Qi L., Liang J. (2010). Interactions between genetic factors that predict diabetes and dietary factors that ultimately impact on risk of diabetes. Curr. Opin. Lipidol..

[48] Yousef A.A., Behiry E.G., Allah W.M.A., Hussien A.M., Abdelmoneam A.A., Imam M.H., Hikal D.M. (2018). IRS-1 genetic polymorphism (r.2963G>A) in type 2 diabetes mellitus patients associated with insulin resistance. Appl. Clin. Genet..

[49] Jia H., Pan Y., Wang Y., Yin F.L., Xu N. (2019). β-3 adrenergic receptor gene polymorphisms are associated with gestational diabetes mellitus in a Chinese population. Medicine.

[50] Liu J., Li J., Li W.J., Wang C.M. (2013). The role of uncoupling proteins in diabetes mellitus. J. Diabetes Res..

[51] Zaccardi F., Webb D.R., Yates T., Davies M.J. (2016). Pathophysiology of type 1 and type 2 diabetes mellitus: A 90-year perspective. Postgrad. Med. J..

[52] Maritim A.C., Sanders R.A., Watkins J.B. (2003). Diabetes, oxidative stress, and antioxidants: A review. J. Biochem. Mol. Toxicol..

[53] Sivitz W.I., Yorek M.A. (2010). Mitochondrial dysfunction in diabetes: From molecular mechanisms to functional significance and therapeutic opportunities. Antioxid Redox Signal..

[54] Lontchi-Yimagou E., Sobngwi E., Matsha T.E., Kengne A.P. (2013). Diabetes mellitus and inflammation. Curr. Diabetes. Rep..

[55] Tan S.Y., Mei Wong J.L., Sim Y.J., Wong S.S., Mohamed Elhassan S.A., Tan S.H., Ling Lim G.P., Rong Tay N.W., Annan N.C., Bhattamisra S.K. (2019). Type 1 and 2 diabetes mellitus: A review on current treatment approach and gene therapy as potential intervention. Diabetes Metab. Syndr..

[56] Usui I., Imamura T., Satoh H., Huang J., Babendure J.L., Hupfeld C.J., Olefsky J.M. (2004). GRK2 is an endogenous protein inhibitor of the insulin signaling pathway for glucose transport stimulation. EMBO J..

[57] Vila-Bedmar R., Cruces-Sande M., Lucas E., Willemen H.L., Heijnen C.J., Kavelaars A., Mayor F., Murga C. (2015). Reversal of diet-induced obesity and insulin resistance by inducible genetic ablation of GRK2. Sci. Signal..

[58] Taguchi K., Hida M., Hasegawa M., Narimatsu H., Matsumoto T., Kobayashi T. (2017). Suppression of GRK2 expression reduces endothelial dysfunction by restoring glucose homeostasis. Sci. Rep..

[59] Cipolletta E., Gambardella J., Fiordelisi A., Del Giudice C., Di Vaia E., Ciccarelli M., Sala M., Campiglia P., Coscioni E., Trimarco B. (2019). Antidiabetic and Cardioprotective Effects of Pharmacological Inhibition of GRK2 in db/db Mice. Int. J. Mol. Sci..

[60] Taguchi K., Sakata K., Ohashi W., Imaizumi T., Imura J., Hattori Y. (2014). Tonic inhibition by G protein-coupled receptor kinase 2 of AKT/endothelial nitric-oxide synthase signaling in human vascular endothelial cells under conditions of hyperglycemia with high insulin levels. J. Pharm. Exp..

[61] Taguchi K., Matsumoto T., Kamata K., Kobayashi T. (2012). G protein-coupled receptor kinase 2, with beta-arrestin 2, impairs insulin-induced AKT/endothelial nitric oxide synthase signaling in ob/ob mouse aorta. Diabetes.

[62] Gao W.Q., Han C.G., Lu X.C., Liu Y.X., Hui H.P., Wang H. (2013). GRK 2 level in peripheral blood lymphocytes of elderly patients with acute myocardial infarction. J. Geriatr. Cardiol..

[63] Iaccarino G., Barbato E., Cipolletta E., De Amicis V., Margulies K.B., Leosco D., Trimarco B., Koch W.J. (2005). Elevated myocardial and lymphocyte GRK2 expression and activity in human heart failure. Eur. Heart J..

[64] Rengo G., Pagano G., Paolillo S., de Lucia C., Femminella G.D., Liccardo D., Cannavo A., Formisano R., Petraglia L., Komici K. (2015). Impact of diabetes mellitus on lymphocyte GRK2 protein levels in patients with heart failure. Eur. J. Clin. Investig..

[65] Lai S., Fu X., Yang S., Zhang S., Lin Q., Zhang M., Chen H. (2020). G protein-coupled receptor kinase-2: A potential biomarker for early diabetic cardiomyopathy. J. Diabetes.

[66] Benjamin E.J., Blaha M.J., Chiuve S.E., Cushman M., Das S.R., Deo R., de Ferranti S.D., Floyd J., Fornage M., Gillespie C. (2017). Heart Disease and Stroke Statistics-2017 Update: A Report From the American Heart Association. Circulation.

[67] Slivnick J., Lampert B.C. (2019). Hypertension and Heart Failure. Heart Fail. Clin..

[68] Landsberg L., Krieger D.R. (1989). Obesity, metabolism, and the sympathetic nervous system. Am. J. Hypertens..

[69] Lucas C.P., Estigarribia J.A., Darga L.L., Reaven G.M. (1985). Insulin and blood pressure in obesity. Hypertension.

[70] Modan M., Halkin H., Almog S., Lusky A., Eshkol A., Shefi M., Shitrit A., Fuchs Z. (1985). Hyperinsulinemia. A link between hypertension obesity and glucose intolerance. J. Clin. Investig..

[71] Ferrannini E., Buzzigoli G., Bonadonna R., Giorico M.A., Oleggini M., Graziadei L., Pedrinelli R., Brandi L., Bevilacqua S. (1987). Insulin resistance in essential hypertension. N. Engl. J. Med..

[72] Sasaki N., Ozono R., Higashi Y., Maeda R., Kihara Y. (2020). Association of Insulin Resistance, Plasma Glucose Level, and Serum Insulin Level With Hypertension in a Population With Different Stages of Impaired Glucose Metabolism. J. Am. Heart Assoc..

[73] Reaven G.M. (2003). Insulin resistance/compensatory hyperinsulinemia, essential hypertension, and cardiovascular disease. J. Clin. Endocrinol. Metab..

[74] Jacobsen P., Rossing K., Hansen B.V., Bie P., Vaag A., Parving H.H. (2003). Effect of short-term hyperglycaemia on haemodynamics in type 1 diabetic patients. J. Intern. Med..

[75] Cleeman J.I., Grundy S.M., Becker D., Clark L. (2001). Expert panel on detection, evaluation and treatment of high blood cholesterol in adults. Executive summary of the third report of the National Cholesterol Education Program (NCEP) Adult Treatment Panel (ATP III). JAMA.

[76] Alberti K.G., Zimmet P.Z. (1998). Definition, diagnosis and classification of diabetes mellitus and its complications. Part 1: Diagnosis and classification of diabetes mellitus provisional report of a WHO consultation. Diabetic Med..

[77] Guerrero-Romero F., Rodríguez-Morán M. (2005). Concordance between the 2005 International Diabetes Federation definition for diagnosing metabolic syndrome with the National Cholesterol Education Program Adult Treatment Panel III and the World Health Organization definitions. Diabetes Care.

[78] De Angelis E., Pecoraro M., Rusciano M.R., Ciccarelli M., Popolo A. (2019). Cross-Talk between Neurohormonal Pathways and the Immune System in Heart Failure: A Review of the Literature. Int. J. Mol. Sci..

[79] Yanai H., Tomono Y., Ito K., Furutani N., Yoshida H., Tada N. (2008). The underlying mechanisms for development of hypertension in the metabolic syndrome. Nutr. J..

[80] Amodio G., Moltedo O., Fasano D., Zerillo L., Oliveti M., Di Pietro P., Faraonio R., Barone P., Pellecchia M.T., De Rosa A. (2019). PERK-Mediated Unfolded Protein Response Activation and Oxidative Stress in PARK20 Fibroblasts. Front. Neurosci..

[81] Gros R., Benovic J.L., Tan C.M., Feldman R.D. (1997). G-protein-coupled receptor kinase activity is increased in hypertension. J. Clin. Investig..

[82] Gros R., Chorazyczewski J., Meek M.D., Benovic J.L., Ferguson S.S.G., Feldman R.D. (2000). G-protein–coupled receptor kinase activity in hypertension: Increased vascular and lymphocyte G-protein receptor kinase-2 protein expression. Hypertension.

[83] Harris D.M., Cohn H.I., Pesant S., Zhou R.H., Eckhart A.D. (2007). Vascular smooth muscle G(q) signaling is involved in high blood pressure in both induced renal and genetic vascular smooth muscle-derived models of hypertension. Am. J. Physiol. Heart Circ. Physiol..

[84] Izzo R., Cipolletta E., Ciccarelli M., Campanile A., Santulli G., Palumbo G., Vasta A., Formisano S., Trimarco B., Iaccarino G. (2008). Enhanced GRK2 expression and desensitization of betaAR vasodilation in hypertensive patients. Clin. Transl. Sci..

[85] Yang J., Villar V.A.M., Armando I., Jose P.A., Zeng C. (2016). G Protein–Coupled Receptor Kinases: Crucial Regulators of Blood Pressure. J. Am. Heart Assoc..

[86] Murga C., Arcones A.C., Cruces-Sande M., Briones A.M., Salaices M., Mayor F. (2019). G Protein-Coupled Receptor Kinase 2 (GRK2) as a Potential Therapeutic Target in Cardiovascular and Metabolic Diseases. Front. Pharm..

[87] Touyz R.M. (2004). Reactive oxygen species, vascular oxidative stress, and redox signaling in hypertension: What is the clinical significance?. Hypertension.

[88] Ciccarelli M., Sorriento D., Franco A., Fusco A., Del Giudice C., Annunziata R., Cipolletta E., Monti M.G., Dorn G.W., Trimarco B. (2013). Endothelial G protein-coupled receptor kinase 2 regulates vascular homeostasis through the control of free radical oxygen species. Arterioscler. Thromb. Vasc. Biol..

[89] Ponikowski P., Voors A.A., Anker S.D., Bueno H., Cleland J.G.F., Coats A.J.S., Falk V., González-Juanatey J.R., Harjola V.-P., Jankowska E.A. (2016). 2016 ESC Guidelines for the diagnosis and treatment of acute and chronic heart failure: The Task Force for the diagnosis and treatment of acute and chronic heart failure of the European Society of Cardiology (ESC)Developed with the special contribution of the Heart Failure Association (HFA) of the ESC. Eur. Heart J..

[90] Cleland J.G.F., Daubert J.-C., Erdmann E., Freemantle N., Gras D., Kappenberger L., Tavazzi L. (2005). The Effect of Cardiac Resynchronization on Morbidity and Mortality in Heart Failure. N. Engl. J. Med..

[91] McMurray J.J.V., Packer M., Desai A.S., Gong J., Lefkowitz M.P., Rizkala A.R., Rouleau J.L., Shi V.C., Solomon S.D., Swedberg K. (2014). Angiotensin–Neprilysin Inhibition versus Enalapril in Heart Failure. N. Engl. J. Med..

[92] Savarese G., Lund L.H. (2017). Global Public Health Burden of Heart Failure. Card. Fail. Rev..

[93] Ibrahim N.E., Januzzi J.L. (2018). Established and Emerging Roles of Biomarkers in Heart Failure. Circ. Res..

[94] Cuomo A., Rodolico A., Galdieri A., Russo M., Campi G., Franco R., Bruno D., Aran L., Carannante A., Attanasio U. (2019). Heart Failure and Cancer: Mechanisms of Old and New Cardiotoxic Drugs in Cancer Patients. Card. Fail. Rev..

[95] Ziaeian B., Fonarow G.C. (2016). Epidemiology and aetiology of heart failure. Nat. Rev. Cardiol..

[96] Braunwald E. (2013). Heart failure. JACC Heart Fail..

[97] Orsborne C., Chaggar P.S., Shaw S.M., Williams S.G. (2017). The renin-angiotensin-aldosterone system in heart failure for the non-specialist: The past, the present and the future. Postgrad. Med. J..

[98] Volpe M., Carnovali M., Mastromarino V. (2016). The natriuretic peptides system in the pathophysiology of heart failure: From molecular basis to treatment. Clin. Sci..

[99] Cohn J.N., Levine T.B., Olivari M.T., Garberg V., Lura D., Francis G.S., Simon A.B., Rector T. (1984). Plasma norepinephrine as a guide to prognosis in patients with chronic congestive heart failure. N. Engl. J. Med..

[100] Sorriento D., Ciccarelli M., Cipolletta E., Trimarco B., Iaccarino G. (2016). “Freeze, Don’t Move”: How to Arrest a Suspect in Heart Failure—A Review on Available GRK2 Inhibitors. Front. Cardiovasc. Med..

[101] Ingwall J.S. (2009). Energy metabolism in heart failure and remodelling. Cardiovasc. Res..

[102] Akhmedov A.T., Rybin V., Marín-García J. (2015). Mitochondrial oxidative metabolism and uncoupling proteins in the failing heart. Heart Fail. Rev..

[103] Woodall M.C., Ciccarelli M., Woodall B.P., Koch W.J. (2014). G protein-coupled receptor kinase 2: A link between myocardial contractile function and cardiac metabolism. Circ. Res..

[104] Premont R.T., Gainetdinov R.R. (2007). Physiological roles of G protein-coupled receptor kinases and arrestins. Annu. Rev. Physiol..

[105] Samuelsson A.M., Bollano E., Mobini R., Larsson B.M., Omerovic E., Fu M., Waagstein F., Holmäng A. (2006). Hyperinsulinemia: Effect on cardiac mass/function, angiotensin II receptor expression, and insulin signaling pathways. Am. J. Physiol. Heart Circ. Physiol..

[106] Fu Q., Xu B., Liu Y., Parikh D., Li J., Li Y., Zhang Y., Riehle C., Zhu Y., Rawlings T. (2014). Insulin inhibits cardiac contractility by inducing a Gi-biased beta2-adrenergic signaling in hearts. Diabetes.

[107] Fu Q., Xu B., Parikh D., Cervantes D., Xiang Y.K. (2015). Insulin induces IRS2-dependent and GRK2-mediated beta2AR internalization to attenuate betaAR signaling in cardiomyocytes. Cell Signal..

[108] Sorriento D., Santulli G., Franco A., Cipolletta E., Napolitano L., Gambardella J., Gomez-Monterrey I., Campiglia P., Trimarco B., Iaccarino G. (2015). Integrating GRK2 and NFkappaB in the Pathophysiology of Cardiac Hypertrophy. J. Cardiovasc. Transl. Res..

[109] Raake P.W., Zhang X., Vinge L.E., Brinks H., Gao E., Jaleel N., Li Y., Tang M., Most P., Dorn G.W. (2012). Cardiac G-protein-coupled receptor kinase 2 ablation induces a novel Ca^2+^ handling phenotype resistant to adverse alterations and remodeling after myocardial infarction. Circulation.

[110] Pfleger J., Gross P., Johnson J., Carter R.L., Gao E., Tilley D.G., Houser S.R., Koch W.J. (2018). G protein-coupled receptor kinase 2 contributes to impaired fatty acid metabolism in the failing heart. J. Mol. Cell. Cardiol..

[111] Sato P.Y., Chuprun J.K., Ibetti J., Cannavo A., Drosatos K., Elrod J.W., Koch W.J. (2015). GRK2 compromises cardiomyocyte mitochondrial function by diminishing fatty acid-mediated oxygen consumption and increasing superoxide levels. J. Mol. Cell. Cardiol..

[112] Sato P.Y., Chuprun J.K., Grisanti L.A., Woodall M.C., Brown B.R., Roy R., Traynham C.J., Ibetti J., Lucchese A.M., Yuan A. (2018). Restricting mitochondrial GRK2 post-ischemia confers cardioprotection by reducing myocyte death and maintaining glucose oxidation. Sci. Signal..

[113] Carotenuto A., Cipolletta E., Gomez-Monterrey I., Sala M., Vernieri E., Limatola A., Bertamino A., Musella S., Sorriento D., Grieco P. (2013). Design, synthesis and efficacy of novel G protein-coupled receptor kinase 2 inhibitors. Eur. J. Med. Chem..

[114] Ciccarelli M., Sorriento D., Fiordelisi A., Gambardella J., Franco A., Del Giudice C., Sala M., Monti M.G., Bertamino A., Campiglia P. (2020). Pharmacological inhibition of GRK2 improves cardiac metabolism and function in experimental heart failure. ESC Heart Fail..

[115] Kashiwagi A., Araki S., Maegawa H. (2021). Sodium-glucose cotransporter 2 inhibitors represent a paradigm shift in the prevention of heart failure in type 2 diabetes patients. J. Diabetes Investig..

[116] Ferrannini E., Mark M., Mayoux E. (2016). CV Protection in the EMPA-REG OUTCOME Trial: A “Thrifty Substrate” Hypothesis. Diabetes Care.

[117] Fields A.V., Patterson B., Karnik A.A., Shannon R.P. (2009). Glucagon-like peptide-1 and myocardial protection: More than glycemic control. Clin. Cardiol..

[118] Gomez-Monterrey I., Carotenuto A., Cipolletta E., Sala M., Vernieri E., Limatola A., Bertamino A., Musella S., Grieco P., Trimarco B. (2014). SAR study and conformational analysis of a series of novel peptide G protein-coupled receptor kinase 2 inhibitors. Biopolymers.

